# 1145. Meningococcal B Immunization for Adolescents in the Presence of Added Gonorrhea Protection: A Public Health Impact Modelling Study in the United States

**DOI:** 10.1093/ofid/ofad500.986

**Published:** 2023-11-27

**Authors:** Zeki Kocaata, Menaka Bhor, Woo-Yun Sohn, Kinga Meszaros

**Affiliations:** GSK, Wavre, Brabant Wallon, Belgium; GSK, Wavre, Brabant Wallon, Belgium; GSK, Wavre, Brabant Wallon, Belgium; GSK, Wavre, Brabant Wallon, Belgium

## Abstract

**Background:**

Several observational studies showed the added gonorrhea protection of outer membrane vesicle (OMV)-containing meningococcal B (MenB) vaccines, raising hopes for achieving WHO gonorrhea control targets. With evolving meningococcal vaccine recommendations in the United States (US), a key policy consideration can be the optimal age for adolescent MenB immunization considering added gonorrhea protection. This study assessed the additional gonorrhea public health impact that could be achieved in the two major existing meningococcal vaccination platforms at 11 and 16-18 years of age in the US.

**Methods:**

A single cohort Markov model was built to simulate primary and recurrent gonorrhea infections (Figure 1). Vaccine effectiveness was assumed to follow recent observational evidence in the US (40% [95% Confidence Interval: 23%, 53%]) post two-doses. Based on clinical evidence on the OMV antigen decay of the 4CMenB vaccine, protection was assumed to wane exponentially over 72 months. The impact was assessed based on (i) uptake rates for completed immunization series (20% to 90%) and (ii) two age groups to mimic the existing MenACWY and MenB vaccination recommendation features for adolescents: 11 vs 17-year-old as a proxy for 16-18-year vaccine recommendation.

**Figure:**
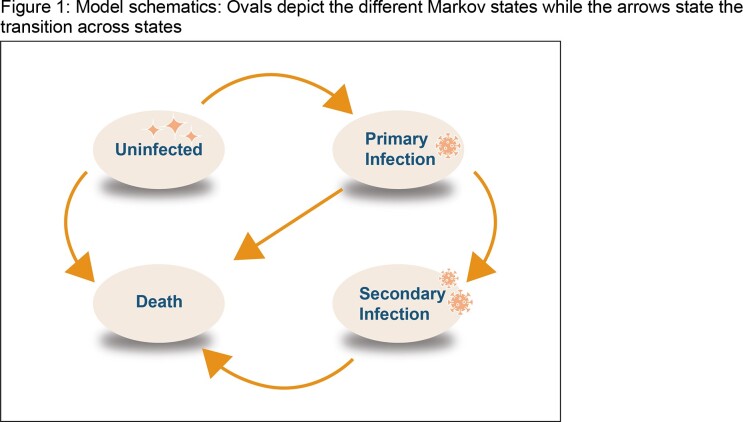

**Results:**

Substantial reductions in gonorrhea prevalence were observed for the cohort following vaccination at age 17 years, ranging from 3.0% [Credibility Interval (CrI): 1.7%, 4.0%] to 13.5% [CrI: 7.8%, 18.0%] for low (20%) to high (90%) uptake scenarios. The reductions in prevalence were substantially lower for vaccination at 11 years of age, ranging from 0.6% [CrI: 0.3%, 0.8%] to 2.7% [CrI: 1.5%, 3.5%] for the same uptake scenarios (Figure 2). Scenario analyses on shorter durations of protection confirmed the directionality of the results (Table 1).

**Figure:**
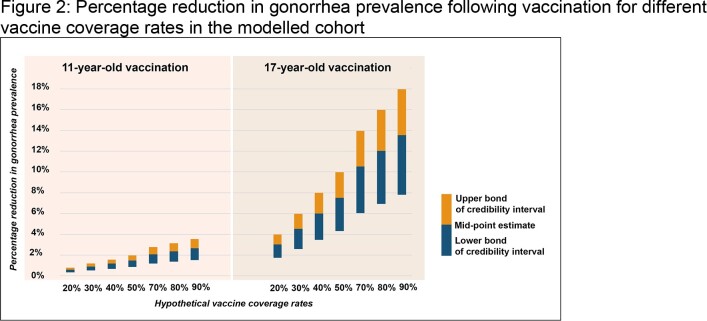

**Figure:**
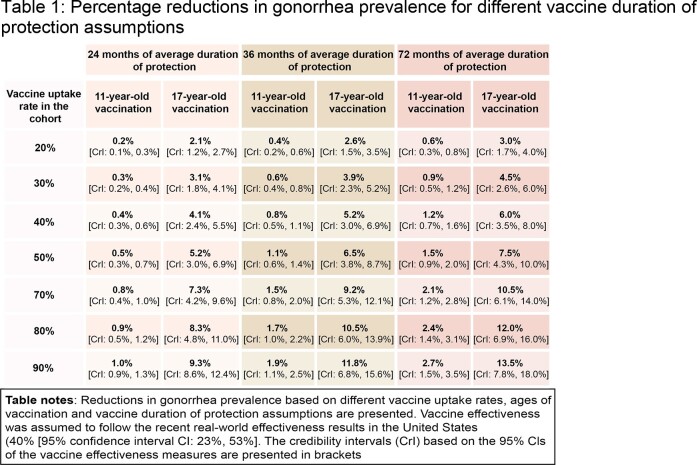

**Conclusion:**

Even with low coverage and without considering disease transmission, an OMV-containing MenB vaccine recommendation for older adolescents could result in significantly higher gonorrhea public health impact compared to younger adolescents. Dual effects for MenB and gonorrhea could be considered when recommending vaccines based on age of vaccination.

**Disclosures:**

**Zeki Kocaata, PhD**, GSK: Stocks/Bonds **Menaka Bhor, PhD**, GSK: Stocks/Bonds **Woo-Yun Sohn, MD**, GSK: Stocks/Bonds **Kinga Meszaros, MBA**, GSK: Employee|GSK: Stocks/Bonds

